# Photoinduced Surface Relief Grating Formation for a Single Crystal of 4-Aminoazobenzene

**DOI:** 10.3390/ijms11041311

**Published:** 2010-03-30

**Authors:** Hideyuki Nakano

**Affiliations:** Department of Applied Chemistry, Faculty of Engineering, Osaka University, Yamadaoka, Suita, Osaka 565-0871, Japan; E-Mail: nakano@chem.eng.osaka-u.ac.jp; Tel.: +81-6-6879-7365; Fax: +81-6-6879-7367

**Keywords:** photoinduced surface relief grating formation, 4-aminoazobenzene, single crystal, photochromism

## Abstract

Photoinduced surface relief grating (SRG) formation for a single crystal of 4-aminoazobenzene was investigated. It was found that SRG could be inscribed on the (001) surface of the crystal, which might suggest that the photoinduced SRG formation is a general phenomenon observed for single crystals of azobenzene-based molecules as well as for azobenzene-based amorphous systems. In addition, the dependences of the SRG formation upon the orientation of the sample crystal and upon the polarization of the writing beams were found to be different from those observed for previously reported crystalline systems.

## 1. Introduction

Patterning and relief fabrication using organic solid materials by photoirradation are key technologies for making micro- and nano-scale eletronic and photonic devices. For example, photolithography using resist materials [1] and laser ablation [2] have been investigated extensively. Photoinduced surface relief grating (SRG) formation by irradiation of the films of azobenzene-based amorphous systems, including polymers [3–12] and small molecules [13–24], with two coherent laser beams, has also received attention as a technique of relief fabrication. Photoinduced SRG formation is believed to be caused by mass transport induced by repetition of trans–cis and cis–trans isomerizations of the azobenzene chromophores. In contrast to such top-down technologies, self-assembly phenomena observed for block copolymers have recently been attracting attention as a bottom-up technology for micro- and nano-fabrications [25–29].

Since the crystallization of small organic molecules is thought to be a kind of self-assembly phenomena, elucidation of the behavior of the molecules at the surface of the crystal may provide valuable information related with the bottom-up technologies using self-assembly phenomena. In addition, it is of interest to fabricate nano-structures on the molecular crystals and to control of the surface structures of the crystals. Very recently, we have demonstrated that photoinduced SRG formation can take place on a single crystal of 4-(dimethylamino)azobenzene (DAAB) and found that the dependence of the SRG formation for the DAAB single crystal upon the polarization of the writing beams was quite different from those reported for azobenzene-based polymers and amorphous molecular materials [30,31]. In addition, the molecules existing in the convex region of the resulting SRG were suggested to be oriented according to the underlying crystal [31]. After that, we have found that SRG formation also took place on a co-crystal of 4-[bis(9,9-dimethylfluoren-2-yl)amino]-azobenzene with ethyl acetate (BFlAB-AcOEt) [32] and on a crystal of 4-[bis(9,9-dimethylfluoren-2-yl)amino]-4′-cyanoazobenzene (CN-BFlAB) [33]. It was suggested that the amorphous layer was produced by photoirradiation of these crystals due to glass-forming ability of BFlAB and CN-BFlAB. As a result, the dependences of the SRG formation for the BFlAB-AcOEt and the CN-BFlAB crystals upon the polarization of the writing beams were similar to those observed for amorphous systems.

**Figure:**
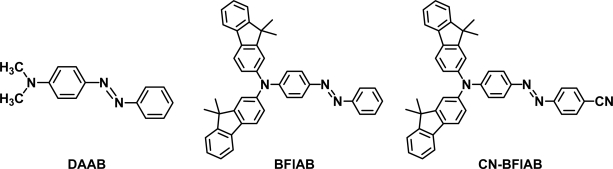


Thus, the photoinduced SRG formation seemed to be a general phenomenon for the single crystals of azobenzen-based molecules as well as for azobenzene-based amorphous systems; however, the study of photoinduced SRG formation for the single crystals is still in an early stage and details regarding the mechanism of SRG formation on the single crystals have not been clear yet. Especially, only DAAB had been found to exhibit SRG formation for the single crystal among azobenzene derivatives without glass-forming ability. In order to gain further information for elucidating the effects of molecular and crystal structures upon the SRG formation and for clarifying the mechanism of the phenomenon, it is necessary to find a variety of organic crystals on which photoinduced SRG formation takes place.

In the present study, a single crystal of 4-aminoazobenzene (AAB) with a good quality for experiments was obtained and then photoinduced SRG formation was investigated using the AAB crystal. AAB has a melting point of 117 °C and no glass-forming ability, exhibiting ready crystallization even when the molten sample was cooled rapidly with liquid nitrogen.

**Figure:**
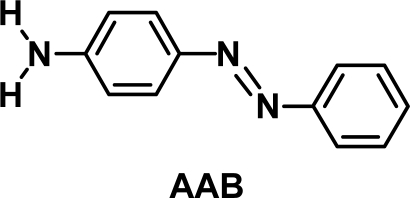


## 2. Results and Discussion

**Figure 1.**
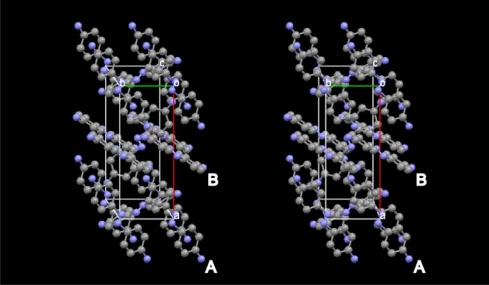
Crystal structure of AAB (stereo view).

A single crystal of AAB with a good quality was obtained as a plate by gradual evaporation of the solvent of the ethanol solution. Then, X-ray crystal structure analysis of the AAB crystal was been performed. It was confirmed that the widest face of the crystal corresponded to (001). Figure 1 shows the crystal structure of AAB, which was quite different from that of DAAB [34]. The crystal included two kinds of non-equivalent AAB molecules (A and B indicated in the Figure 1). A half of each molecule was independent, and both A and B molecules possessed the inversion centers at the centers of their N=N bonds. Thus, amino-groups were disordered, existing at either end of the azobenzene moiety. The AAB molecules were oriented in a variety of directions in the crystal, therefore the optical anisotropy of the crystal was rather small. Assuming the transition moment of the AAB molecule was parallel to the line connecting 4-carbon with 4′-carbon of the azobenzene moiety, the dichroic ratio for the (001) surface of the AAB crystal, being defined here as the ratio of the optical density for polarized light parallel to the b-axis to that parallel to the a-axis, was estimated to be *circa* 1.1, which was considerably smaller than those for the (001) and (100) surfaces of DAAB crystal (*circa* 17 and 106, respectively) [31].

**Figure 2.**
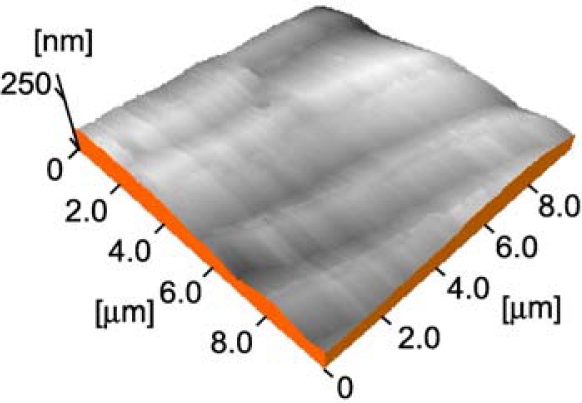
Typical AFM image of the (001) surface of AAB single crystal before photoirradiation.

The (001) surface of the AAB single crystal was found to be fairly but not completely flat. The typical structure of the surface is shown in Figure 2, showing that ridges and troughs exist with a level of *circa* 10–30 nm. Some other defects like holes and textured surfaces with a level of *circa*10–30 nm were observed in some locations. Such defects of the surface may be caused by the fragility of the surface of the AAB crystal.

**Figure 3.**
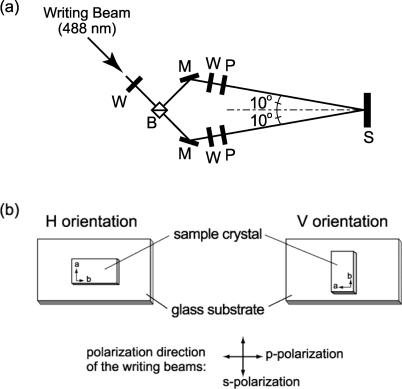
(**a**) Schematic experimental setup for photoinduced SRG formation. S: sample, P: polarizer, W: wave plate, M: mirror, B: beam splitter. (**b**) Schematic illustration of the sample orientation.

Using the AAB single crystal, photoinduced SRG formation was investigated. Schematic experimental setup for photoinduced SRG formation is illustrated in Figure 3a. Two coherent laser beams (488 nm, 5 mW × 2) with either p-polarization or s-polarization were used as writing beams that irradiated the (001) surface of the crystal at room temperature. Under these conditions, the period of the interference pattern is 1.41 μm. The sample crystal was fixed on a glass substrate to be oriented as the b-axis of the crystal [35], almost parallel to the polarization plane of either p- or s-polarization of the writing laser beams, which were referred to here as H orientation or V orientation, respectively (Figure 2b).

**Figure 4.**
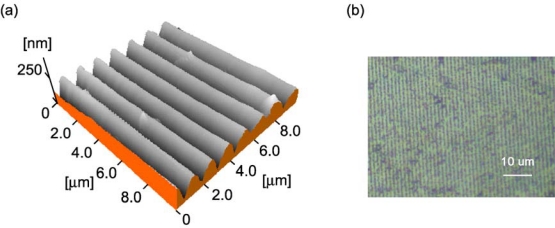
(**a**) AFM and (**b**) optical images of SRG inscribed on AAB single crystal.

SRG formation was found to take place on the AAB single crystal by interference irradiation with the writing beams, confirmed by atomic force microscopy (AFM) and optical microscopy. Figure 4a shows an AFM image of the surface of the H-oriented sample after irradiation with the s-polarized writing beams for 20 min. The SRG with a modulation depth of *circa* 150 nm was observed. As shown in Figure 4b, the optical microscopy also confirmed that the SRG was inscribed in the wide area with a interval of *circa* 1.4 μm, which is identical to the predicted interval of the interference pattern produced under the conditions as shown in Figure 3a. The resulting SRG was fairly stable, with no significant deformation of the resulting SRG observed at room temperature for several days.

Like previously reported crystals, photoinduced SRG formation for the AAB crystal was found to depend upon both the orientation of the sample crystal and the polarization of the writing beams. However, the dependences were different from those observed for the previously reported crystals [30–32]. Table 1 summarizes the modulation depths of the SRGs inscribed on the AAB crystal by irradiation with the writing beams for 20 min under different experimental conditions. We have reported that the dependences of the SRG formation for DAAB and BFlAB-AcOEt crystals upon the orientation of the sample were due to large optical anisotropy of the sample crystal [30–32]. On the other hand, the SRG formation was independent of the orientation of the sample for the CN-BFlAB crystal of which the optical anisotropy was small. In the present study; however, the SRG formation for the AAB crystal was found to depend upon the orientation of the sample crystal even though the optical anisotropy for the crystal was quite small as described above. That is, by using the p-polarized writing beams, a SRG with a larger modulation depth was obtained for the V-oriented sample (Entry No.2) than for the H-oriented sample (No.1). Similarly, the modulation depth of resulting SRG was larger for the H-oriented sample (No.3) than for the V-oriented one (No.4) when the s-polarized writing beams were used. With regard to the dependence of the SRG formation upon the polarization of the writing beams, we have reported that the s-polarized beams were preferable for the SRG formation for the DAAB single crystal relative to the p-polarized beams [30,31]. However, that is not necessarily so for the AAB crystal. That is, the p-polarized beams were preferable for the V-oriented sample whereas the s-polarized beams were preferable for the H-oriented one. Although the distribution of the modulation depth of resulting SRG seemed to be somewhat large maybe due to the fragility of the surface of the AAB crystal, the result indicated that the writing beams with the polarization direction parallel to the a-axis of the crystal (No. 2 and 3) could inscribe larger SRG than those parallel to the b-axis (No. 1 and 4).

The results seem to be due to the existence of two kinds of molecules (A and B indicated in Figure 1) in the crystal. Because of their different molecular orientations in the crystal, the absorption of the incident polarized writing beams near the surface of the crystal by the A molecules was different from that by the B molecules. Table 2 summarizes the estimated relative molar extinction coefficients of the molecules A and B in the crystal for the polarized beams traveling in the direction normal to the (001) surface, where the transition moment of the AAB molecule was assumed to be parallel to the line connecting 4-carbon with 4′-carbon of the azobenzene moiety. The result indicated that the incident beams with polarization direction parallel to the a-axis was absorbed mainly by the A molecules while the absorption of the incident beams with the polarization direction parallel to the b-axis by the A molecules was comparable to that by the B molecules. Thus, it can be assumed that the A molecules activated by absorbing the irradiated beam are mobile more effectively to inscribe SRG than the activated B molecules. That is, the A molecules were more preferentially activated by the writing beams with polarization direction parallel to a-axis (No. 2 and 3) than those parallel to b-axis (No. 1 and 4), and hence the SRG with larger modulation depth was inscribed under the conditions No. 2 and 3 than under the conditions No. 1 and 4. Figure 1 shows that the A molecules face in the different direction from the B molecules. Such a difference may cause the difference in mobility of the activated molecules. Thus, the present study revealed that the crystal structure, namely molecular orientation in the crystal, is one of the important factors for determining the SRG-forming characteristics for the single crystals.

## 3. Experimental Section

Single crystal of AAB was obtained by gradual evaporation of the solvent of ethanol solution of AAB. X-Ray structure analysis was performed using a single crystal with dimensions of approximately 0.40 × 0.40 × 0.50 mm^3^ on a Rigaku RAXIS-RAPID Imaging Plate diffractometer with graphite-monochromated Mo-*K*α (0.71069 Å) radiation. A total of 21592 independent reflections was obtained of which 3245 were unique. The structure was solved by direct methods and refined by full matrix least square method. Crystallographic data were as follows: C_12_H_11_N_3_, M = 197.24, monoclinic, space group *P*2_1_/n, a = 13.750(1), b = 5.6253(4), c = 14.023(1)Å, β = 98.400(2)°, V = 1073.1(1)Å^3^, Z = 4, Dc = 1.221 g cm^−1^, F_000_ = 416.0, μ(MoKα) = 0.76 cm^−1^, T = 25 °C, *R*_1_[I > 2σ(I)] = 0.091, *wR*_2_ = 0.221. Crystallographic data (excluding structure factors) for AAB have been deposited with the Cambridge Crystallographic Data Centre as supplementary publication nos. CCDC-761536. Copies of the data can be obtained free of charge on application to CCDC, 12 Union Road, Cambridge CB2 1EZ, UK [Fax: (internat.) +44-1223/336-033; E-Mail: deposit@ccdc.cam.ac.uk].

Photoinduced SRG formation was carried out by using a compact CW laser (488 nm: CYAN-488-50NH-W, Spectra Physics) as a source of writing beams at room temperature. The sample crystal with a thickness of 0.2–1.0 mm was fixed on a glass substrate (Micro Slide Glass, Matsunami Glass Ind., Ltd.) by an epoxy-type adhesive agent. Formation of SRG was confirmed by means of Scanning Probe Microscope (JSTM-4200D, JEOL Ltd.) with a micro cantilever (OMCL-AC160T-C2, OLYMPUS) and an Optiphot X2 (Nikon) microscope.

## 4. Calculation

Relative molar extinction coefficients of the molecules A and B in the crystal for the incident beams with polarization directions parallel to the a-axis (ɛ^rel^_a_) and the b-axis (ɛ^rel^_b_) traveling in the direction normal to the (001) surface was estimated as follows. The crystal structure analysis of AAB indicated that the coordination factors of 4-carbon of azobenzene moieties were (0.2559, −0.4361, 1.1440) and (0.3683, 0.4863, 0.7591) for the molecules A and B, respectively. By symmetry operation, the factors of 4′-carbons were determined to be (−0.2559, 0.4361, 0.8560) and (0.6317, −0.4863, 1.2409). Using these values and unit cell parameters, the unit vectors parallel to the line connecting 4-carbon with 4′-carbon of the azobenzene moiety could be determined to be (0.7137, −0.5431, 0.4423) for the molecule A and (0.2904, −0.6060, 0.7405) for the molecule B. Assuming that these unit vectors were parallel to the transition moments of the corresponding molecules A and B, the values ɛ^rel^_a_ and ɛ^rel^_b_ could be estimated to be squares of the x- and y-components of their unit vectors, respectively, as summarized in Table 2.

Dichroic ratio for the (001) surface of the crystal was estimated to be the ratio of the sum of the ɛ^rel^_b_ values for the molecules A and B to the sum of the ɛ^rel^_a_ values for the molecules A and B.

## 5. Conclusions

Photoinduced SRG formation was observed to take place at the (001) surface of the AAB single crystal of AAB in the present study. This fact might suggest that the photoinduced SRG formation is a general phenomenon for single crystals of azobenzene-based molecules, as well as for azobenzene-based amorphous polymers and amorphous molecular materials. In addition, the dependences of the SRG formation for the AAB single crystal upon the orientation of the sample crystal and upon the polarization of the writing beams were different from those observed for previously reported crystalline systems. Although the detailed mechanism of the SRG formation has not been clear yet, it is suggested that the crystal structures play an important role for the photoinduced SRG formation for the single crystals.

## Figures and Tables

**Table 1.** Modulation depth of SRG inscribed on the AAB crystal by irradiation with writing beams (5 mW × 2) for 20 min.

**Entry**	**Polarization of the writing beams**	**Orientation of the sample crystal**	**Modulation depth [nm]**
1	p	H	30–70
2	p	V	80–250
3	s	H	100–200
4	s	V	40–80

**Table 2.** Estimated relative molar extinction coefficients of the molecules A and B in the crystal for the incident beams with polarization directions parallel to the a-axis (ɛ^rel^_a_) and the b-axis (ɛ^rel^_b_) traveling in the direction normal to the (001) surface.

**Molecule**	**ɛ^rel^_a_**	**ɛ^rel^_b_**
A	0.51	0.29
B	0.08	0.37
